# Psychopharmacological Treatment in Patients with Heart Failure: A Narrative Review of Mood Stabilizers and Antipsychotics

**DOI:** 10.3390/brainsci16050452

**Published:** 2026-04-24

**Authors:** Błażej Pilarski, Szymon Florek, Alexander Suchodolski, Mariola Szulik, Robert Pudlo

**Affiliations:** 1 Doctoral School, Department of Psychoprophylaxis, Faculty of Medical Sciences in Zabrze, Medical University of Silesia in Katowice, 42-612 Tarnowskie Góry, Poland; d201331@365.sum.edu.pl; 2 Department of Psychoprophylaxis, Faculty of Medical Sciences in Zabrze, Medical University of Silesia in Katowice, 42-612 Tarnowskie Góry, Poland; rpudlo@sum.edu.pl; 3 Department of Cardiology and Electrotherapy, Silesian Center for Heart Diseases, Faculty of Medical Sciences in Zabrze, Medical University of Silesia, Katowice, 41-800 Zabrze, Poland; alex.suchodolski@gmail.com; 4 Department of Medical Sciences, Collegium Medicum, WSB University, 41-300 Dabrowa Górnicza, Poland; mszulik3@wp.pl

**Keywords:** heart failure, psychopharmacology, mood stabilizers, lithium, antipsychotics, drug interactions, QT prolongation, polypharmacy, cardiotoxicity, psychiatric comorbidity

## Abstract

**Highlights:**

**What are the main findings?**

- Patients with HF and psychiatric comorbidity are at increased risk of clinically significant drug–drug interactions due to unavoidable polypharmacy; lithium has a narrow therapeutic index and is highly vulnerable to interactions with HF therapies that affect renal sodium handling (RAAS inhibitors, diuretics, SGLT2 inhibitors).
- Antipsychotic drugs differ substantially in their arrhythmic and hemodynamic risk profiles; QTc prolongation, orthostatic hypotension, and electrolyte disturbances require agent-specific assessment in patients with HF.

**What are the implications of the main findings?**

- Safe management requires close collaboration between cardiology and psychiatry, individualized risk stratification, and regular laboratory and electrocardiographic monitoring.
- There is a critical need for prospective studies and clearer guideline integration addressing combined cardiologic and psychotropic pharmacotherapy in HF.

**Abstract:**

Heart failure (HF) is a leading cause of morbidity and mortality worldwide, and its co-occurrence with psychiatric disorders poses significant therapeutic challenges. This narrative review examines the safe use of psychopharmacological agents in patients with HF, focusing on mood stabilizers (particularly lithium) and antipsychotics. We summarize clinically relevant pharmacokinetic and pharmacodynamic interactions between these agents and guideline-directed HF therapies, including ACEIs, ARBs, ARNIs, beta-blockers, MRAs, SGLT2 inhibitors, and diuretics. Lithium is particularly prone to interactions due to its narrow therapeutic index and dependence on renal sodium handling, with RAAS inhibitors, thiazide diuretics, and SGLT2 inhibitors increasing the risk of toxicity. Antipsychotics are associated with QT prolongation, orthostatic hypotension, and electrolyte disturbances, with substantial variability between agents. This review highlights key clinical risks, provides a practical summary of drug interactions, and outlines principles for individualized, multidisciplinary management. Care requires coordinated cardiology–psychiatry collaboration, careful drug selection, and assessment of renal function, electrolytes, drug levels, and ECG parameters. Further studies and improved guideline integration are needed.

## 1. Introduction

### Background on Heart Failure (HF)

In view of significant advances in cardiological diagnostics and therapy, heart failure (HF) remains one of the leading causes of morbidity and mortality worldwide, both in developed and developing countries. According to the latest data, the global prevalence of HF reaches approximately 682.7 per 100,000 people, with higher rates observed in men (760.8/100,000) than in women (604.0/100,000) [1]. In the European population, it is estimated that the disease affects about 1–2% of adults, and after the age of 70 even more than 10% of the population [2]. It is worth emphasizing that this problem does not concern only the classical form of HF with reduced left ventricular ejection fraction (HFrEF), but also patients with preserved (HFpEF) and mildly reduced/intermediate (HFmrEF) ejection fraction, in whom the prognosis remains unfavorable, although it shows significant prognostic and therapeutic differences compared with HFrEF [3].

The manuscript is structured according to classes of heart failure pharmacotherapy to reflect the framework of guideline-directed medical treatment. While this approach may require cross-referencing from a psychopharmacological perspective, it allows interactions to be interpreted within the clinical context in which they most frequently arise.

The co-occurrence of mental disorders in patients with HF constitutes a serious and often underestimated clinical problem. HF, as a chronic disease, predisposes patients to the development of mood disorders and psychotic disorders through both biological mechanisms—such as activation of the hypothalamic–pituitary–adrenal axis, inflammatory processes, or cerebral hypoperfusion—and psychosocial factors, including deterioration in quality of life, functional limitations, recurrent hospitalizations, and fear of disease exacerbation. Additionally, HF and mental disorders often share common risk factors, such as arterial hypertension, coronary artery disease, diabetes mellitus, and smoking [4,5]. Patients with such comorbidity represent a particular therapeutic challenge for both cardiologists and psychiatrists, as polypharmacotherapy increases the risk of interactions between cardiovascular and psychotropic medications (Table 1), affecting treatment effectiveness and patient safety. This is particularly relevant in light of recent European Society of Cardiology (ESC) guidance emphasizing the need for integrated cardiovascular and mental health care, especially in patients exposed to polypharmacy, arrhythmic risk, and renal or electrolyte disturbances. Accordingly, the present review is intended not only as a pharmacological overview, but also as a clinically oriented synthesis aligned with current heart failure management principles [6].

Contemporary pharmacotherapy of HF is based on several key therapeutic pillars with proven effects on survival and reduction in hospitalization risk. These include angiotensin-converting enzyme inhibitors (ACEIs), angiotensin receptor blockers (ARBs), angiotensin receptor–neprilysin inhibitors (ARNIs), beta-blockers, mineralocorticoid receptor antagonists (MRAs), and SGLT2 inhibitors. Although diuretics do not affect mortality, they remain a crucial component of symptomatic treatment and are widely used in clinical practice [7,8,9]. At the same time, the number of patients with HF requiring psychiatric care is increasing, which further complicates therapeutic management. In this review article, we focus on two key groups of psychotropic medications in this population: mood stabilizers, with particular emphasis on lithium, and antipsychotic drugs, both typical and atypical. Treatment with antipsychotic medications of both the first generation (FGA, First-Generation Antipsychotics) and the second generation (SGA, Second-Generation Antipsychotics) is associated with a significantly higher risk of sudden cardiac death compared with individuals not receiving these medications, and this risk appears to be dose-dependent. For both classes of drugs, higher doses were associated with higher incidence rates, which for high doses were 2.42 for typical antipsychotics and 2.86 for atypical antipsychotics, respectively. However, no significantly increased risk was observed in individuals who had used antipsychotic treatment in the past [10].

The aim of this study is to present current data on potential pharmacological interactions between standard HF therapy and selected groups of psychotropic medications (i.e., mood stabilizers, with particular emphasis on lithium, as well as typical and atypical antipsychotics). The article identifies the most important clinical risks and formulates practical conclusions to support the clinical decision-making process. This work represents an attempt to synthesize the currently dispersed knowledge and to create a practical guide for clinicians managing patients with HF and coexisting mental disorders, with particular emphasis placed on the clinical significance of the interactions discussed.

This review is part of a larger project in which we aim to discuss the interactions between psychotropic drugs and cardiovascular medications in patients with HF. In this review, we have focused on mood stabilizers and antipsychotics, as they raise particularly significant concerns regarding the risk of arrhythmias, hemodynamic effects, renal clearance, and drug–drug interactions in patients with heart failure. Antidepressants and anxiolytics will be discussed in a separate study.

## 2. Methodology

This narrative review was based on a targeted, problem-oriented literature search conducted in major biomedical databases, including PubMed/MEDLINE, Scopus, and Google Scholar, as well as selected guidelines and regulatory documents. The search covered publications from 1987 to 2025, with particular emphasis on studies published between 2020 and 2025. Literature was selected based on its relevance to the review topic, with priority given to clinical guidelines, systematic reviews, meta-analyses, randomized clinical trials, pharmacological studies, and reports addressing drug interactions and cardiovascular safety in psychiatric treatment. Older landmark studies were also included where they provided important mechanistic, pharmacokinetic, or historical context.

The reviewed evidence was interpreted according to study design and level of clinical applicability. Preclinical studies, case reports, observational studies, and clinical trials were considered separately to avoid overinterpretation of lower-level evidence.

## 3. Angiotensin-Converting Enzyme Inhibitors (ACEIs), Angiotensin Receptor Blockers (ARBs), and ARNIs (Angiotensin Receptor–Neprilysin Inhibitors—Sacubitril/Valsartan)

ACEIs are a key class of drugs used in the treatment of arterial hypertension and in the prevention and management of cardiovascular complications such as congestive HF, coronary artery disease, myocardial infarction, and chronic kidney disease. Their mechanism of action involves the competitive inhibition of the angiotensin-converting enzyme (ACE), which blocks the conversion of angiotensin I into the vasoconstrictive angiotensin II, leading to inhibition of the renin–angiotensin–aldosterone system (RAAS) [11].

ARBs are effective and well-tolerated antihypertensive agents as well as drugs used in the treatment of HF. They act by selectively blocking the AT1 receptor, which may lead to a reduction in peripheral vascular resistance. ARBs exhibit cardio-and nephroprotective effects, reducing left ventricular hypertrophy and microalbuminuria, while maintaining a low incidence of adverse effects. Differences in their molecular structure result in diverse pharmacokinetic profiles and receptor affinities, which influence the duration and magnitude of their antihypertensive action [12]. ARBs in HF are used when ACE inhibitors cause adverse effects (e.g., dry cough) or are not tolerated by the patient. Sacubitril/valsartan is a combination of a neprilysin inhibitor (sacubitril) and an ARBs (valsartan), used in the treatment of HF with reduced ejection fraction [13]. The pharmacokinetics of the drug are characterized by rapid absorption and conversion of the prodrug (sacubitril) into its active metabolite, sacubitrilat, whose exposure is significantly increased in patients with HF and correlates with the degree of renal impairment. The clearance of sacubitrilat depends on renal function, whereas the elimination of valsartan occurs mainly via the biliary route, which means that moderate hepatic impairment significantly increases its systemic exposure [14]. ACEIs or ARBs are used in the treatment of HF in cases of intolerance to or unavailability of ARNI therapy, which currently represents the preferred treatment option in patients with HFrEF. However, they should not be used concomitantly due to the increased risk of renal impairment and hyperkalemia [2].

### 3.1. Interactions of Mood Stabilizers with ACEIs, ARBs, and ARNIs in the Treatment of HF (Table 1)

#### 3.1.1. Lithium and ACEIs

The mechanism of action of lithium ions (Li^+^) is primarily based on the displacement of sodium (Na^+^) and potassium (K^+^) ions during their transport across the cell membranes of neurons and cardiomyocytes. This may lead to stabilization of cell membranes, reduced neuronal excitability, and decreased synaptic transmission. Regular assessment of serum lithium concentrations is required and should be performed under standardized conditions that allow the results to be interpreted in relation to established therapeutic ranges. Lithium therapy also requires periodic monitoring of electrolyte levels, particularly sodium, as well as renal function parameters, especially in situations that may predispose to disturbances in fluid and electrolyte balance (e.g., during infections). It is also important to ensure adequate dietary sodium intake, because lithium inhibits the reabsorption of sodium ions in the renal tubules, and sodium deficiency may lead to increased lithium retention and a higher risk of toxic effects [15].

A retrospective case–control study (*N* = 20) demonstrated that ACE inhibitors increase steady-state lithium concentrations by 36.1% and reduce its clearance by 25.5% (*p* < 0.0001), with 20% of patients developing symptoms of toxicity. Correlation analysis revealed significant associations between reduced lithium clearance and age (*r* = −0.45; *p* < 0.05), as well as serum creatinine concentration (*r* = −0.52; *p* < 0.02). Multiple regression analysis showed that approximately 25% of the variability in lithium clearance could be explained by changes in serum creatinine levels, and inclusion of age in the model increased this proportion to 35%. The authors of the cited study suggest that the concomitant use of lithium and ACE inhibitors should be avoided, particularly in older patients with reduced renal reserve [16]; however, in excellent lithium responders and in individuals at high risk of suicide, it is generally recommended to reduce the lithium dose and closely monitor serum lithium levels, particularly during changes in the patient’s somatic condition, such as infection or malnutrition.

In clinical practice, the combination of lithium with ACE inhibitors may be considered in patients who develop hypertension during long-term lithium therapy; however, this combination has been associated with an increased risk of lithium toxicity. Three cases of interactions with enalapril, lisinopril, and ramipril have been described, in which the key risk factors for toxicity may have been the concomitant use of hydrochlorothiazide or other diuretics, rather than the ACEI–lithium combination itself. These findings suggest that, with appropriate precautions—including regular monitoring of lithium levels, maintaining adequate hydration, and avoiding diuretics—such a combination may be used in selected cases [17]. ACEIs, such as enalapril, may reduce the renal clearance of lithium, leading to an increase in its serum concentration and a higher risk of toxicity. Reports, including a case study describing a 31% increase in lithium concentration, confirm the clinical significance of this interaction [11]. In patients with HF, who are particularly susceptible to electrolyte disturbances, this risk is further increased. The probability and severity of the interaction depend on the dose of the ACE inhibitor, the baseline lithium concentration, and renal function. In clinical practice, close clinical observation with repeated lithium level assessment is required, especially during the initiation or dose adjustment of ACE inhibitor therapy [18]. After 9 years of stable therapy with lithium and an ACE inhibitor, a sudden and severe lithium toxicity occurred, presenting with delirium and acute renal failure, despite regular monitoring of lithium levels. This case highlights that this interaction may manifest in a delayed and abrupt manner. Therefore, continued vigilance is required even in clinically stable patients, and potential triggering factors—such as infection or deterioration of renal function—should always be taken into account [19].

ACE inhibitors and ARBs may increase serum lithium concentrations, thereby raising the risk of toxicity. Therefore, in patients who are initiating or discontinuing therapy with these agents, close monitoring of serum lithium levels is essential. In such situations, the lithium dose often needs to be reduced until its therapeutic concentration is re-established and stabilized [20].

#### 3.1.2. Lithium and ARBs

The use of ARBs has been reported with a significant risk of interaction with lithium, leading to an increase in serum lithium concentration and consequently a higher likelihood of toxic effects. This phenomenon mainly results from a reduction in the renal clearance of lithium due to the effect of ARBs on transport processes in the proximal renal tubules. In clinical practice, this requires particularly careful monitoring of lithium serum levels after initiation of ARB therapy or changes in its dosage, and, if necessary, appropriate adjustment of the lithium dose. Furthermore, ARB therapy requires routine monitoring for other potential adverse effects, including hypotension, deterioration of renal function, and hyperkalemia. It is recommended to monitor serum creatinine, potassium, sodium levels, and estimated glomerular filtration rate (eGFR). In patients with HF, regular assessment of left ventricular ejection fraction (LVEF) is also necessary [21]. In patients with HF, in whom ARBs are often used concomitantly with lithium, an additional component of monitoring is the assessment of left ventricular ejection fraction. In this group of patients, the risk of lithium accumulation increases, because ARBs may induce sodium loss, and the resulting secondary hyponatremia enhances renal lithium reabsorption. The situation may be further aggravated by dehydration or progressive renal dysfunction, which can lead to a dangerous increase in serum lithium levels. Therefore, in patients receiving both medications, closer surveillance of renal parameters and electrolytes, particularly sodium, is required, along with regular assessment of serum lithium concentrations [22].

#### 3.1.3. Lithium and ARNI

Despite the established role of sacubitril/valsartan in the treatment of HF, its interaction with lithium remains poorly understood. There are no dedicated large randomized studies that have prospectively assessed the safety of this combination. The available evidence comes mainly from retrospective analyses, regulatory agency communications, and individual case reports concerning the effects of ARNI on the RAAS. These reports indicate that the use of these agents may lead to a reversible increase in serum lithium concentrations and the occurrence of symptoms of lithium toxicity, which is particularly relevant in cardiological patients, who often present with a risk of impaired renal function. Therefore, the routine co-administration of sacubitril/valsartan and lithium should be avoided in patients with HF. If such a combination is considered necessary, regular lithium level assessment and careful observation for signs of toxicity are required, with particular caution in patients receiving diuretics due to the increased risk of nephrotoxicity [23]. The combination of sacubitril and valsartan may lead to an increase in serum lithium concentration and thereby increase the risk of lithium toxicity. Sacubitril (in combination with valsartan) inhibits the RAAS, which may impair the renal excretion of lithium. Reduced sodium reabsorption in the renal tubules may result in increased lithium retention [24].

#### 3.1.4. Antiepileptic Drugs and ACEIs

An animal model study demonstrated that the combination of ACE inhibitors (enalapril, cilazapril) with carbamazepine (CBZ) or valproate (VPA) is safe in terms of interactions that could reduce anticonvulsant efficacy. Moreover, enalapril significantly enhanced the anticonvulsant effect of VPA, which may have potential clinical relevance. No adverse effects on cognitive or motor functions were observed with the combined treatment [25].

#### 3.1.5. Lamotrigine and ACEIs

One study demonstrated a significant pharmacodynamic interaction between the ACE inhibitor enalapril and lamotrigine (LTG). In the maximal electroshock (MES) model in mice, enalapril at a dose of 30 mg/kg significantly (*p* < 0.01) enhanced the anticonvulsant effect of LTG, reducing its effective dose (ED50) from 5.3 mg/kg to 3.6 mg/kg. The interaction appears to be pharmacodynamic in nature, as enalapril did not alter LTG concentrations in plasma or in the brain. In contrast to enalapril, cilazapril did not affect the activity of LTG. These findings suggest that enalapril may potentially enhance the efficacy of LTG in the treatment of tonic–clonic seizures, without evidence of adverse pharmacokinetic interactions [25]. Recent FDA communications have highlighted that LTG, when used as a mood stabilizer, may carry an increased risk of serious arrhythmias in patients with HF. The primary mechanism of the potential pharmacodynamic interaction involves proarrhythmic effects of LTG that—as suggested by in vitro studies—may result from blockade of sodium channels in cardiomyocytes, potentially acting synergistically with pre-existing electrical instability of the myocardium in HF, and further potentiated by electrolyte disturbances frequently encountered in this disease (e.g., hypokalemia). Additionally, the primary concern relates to the 2-N-methyl metabolite of LTG, which may cause dose-dependent atrioventricular conduction disturbances, including PR prolongation, QRS widening, and complete AV block [26,27]. HF, through its impact on hepatic and renal function, may also alter the metabolism and clearance of LTG, potentially leading to higher-than-expected drug concentrations. Prior to initiating LTG in a patient with HF, a thorough cardiological assessment encompassing the full treatment regimen is therefore essential, including regular ECG evaluation and serum electrolyte measurement.

#### 3.1.6. Comparison of Anticonvulsant Drugs and LTG in the Context of Their Co-Administration with ACEIs

In a retrospective cohort study using Danish national registries, involving older patients (≥65 years) with concomitant HF and epilepsy, the use of VPA was associated with a significantly higher risk of mortality compared with treatment using levetiracetam (LEV) or LTG. In the adjusted analysis, patients treated with VPA had a 37% higher risk of all-cause mortality (HR 1.37; 95% CI 1.01–1.85) and more than a twofold higher risk of death due to HF (HR 2.39; 95% CI 1.02–5.60). These findings suggest that in elderly patients with HF, LEV or LTG may represent safer therapeutic alternatives to VPA, when clinically appropriate [28].

In the available scientific literature, information regarding possible interactions between widely used psychiatric mood stabilizers—carbamazepine, lamotrigine, and valproic acid—and cardiovascular drugs from the ACEI, ARB, and ARNI classes in patients with HF is significantly limited, which makes it difficult to fully assess the risks associated with such therapeutic combinations.

### 3.2. Interactions of Antipsychotic Drugs with ACEIs, ARBs, and ARNIs (Table 1)

Antipsychotic drugs are primarily used in the treatment of schizophrenia and other psychotic disorders. They are also used in manic episodes in bipolar disorder, in the short-term management of disturbances of consciousness, including agitation and aggression, and SGAs may be used as adjunctive therapy in severe depression with psychotic features [29,30,31,32]. The classical classification of antipsychotics distinguishes between “typical” (first-generation) agents—such as haloperidol, chlorpromazine, and perphenazine—and “atypical” (second-generation) agents, including olanzapine, risperidone, quetiapine, aripiprazole, and clozapine. The choice of a specific antipsychotic depends on the clinical presentation, previous treatment response, the adverse effect profile, and the presence of comorbid conditions [29,30,31,32,33].

In an analysis including 2493 cases and 7478 controls, the use of antipsychotic medications was associated with an increased risk of cardiovascular diseases (odds ratio [OR] = 1.54; 95% CI: 1.32–1.79). In particular, a significant increase in the risk of ischemic HF was observed (OR = 2.26; 95% CI: 1.71–2.99). Among the drugs significantly associated with increased cardiovascular risk were haloperidol, aripiprazole, quetiapine, olanzapine, risperidone, sulpiride, and chlorpromazine. A dose–response analysis revealed a nonlinear relationship, with the greatest increase in risk observed at lower doses, while at higher doses the trend stabilized. These findings suggest that in patients with schizophrenia, the use of antipsychotic medications may significantly increase the risk of cardiovascular disease, and that the magnitude of this risk depends both on the specific drug used and on the characteristics of the underlying disease [34].

Orthostatic hypotension is one of the most common cardiovascular adverse effects of antipsychotic medications, with a higher incidence observed in elderly patients. When combined with ACEIs, ARBs, or ARNIs, this effect may lead to clinically significant orthostatic hypotension. In a study involving 5201 patients aged over 65 years, asymptomatic orthostatic hypotension was observed in 16.2–18% of participants, whereas symptomatic cases occurred in only 2%. Among first-generation antipsychotics (FGAs), low-potency phenothiazines, such as chlorpromazine and thioridazine, are widely recognized as major contributors to orthostatic hypotension [35].

Very rare cases of psychiatric symptoms have been described in the literature among patients treated with sacubitril/valsartan, although the causal relationship remains unclear. The use of sacubitril/valsartan has been associated with symptoms such as hallucinations, paranoid delusions, and sleep disturbances, which usually occurred within 2–7 days after initiation of treatment or dose escalation. The mechanism underlying this phenomenon has not yet been clearly explained; however, it has been suggested that inhibition of neprilysin activity may reduce the degradation of beta-amyloid, a protein associated with the pathogenesis of neurodegenerative diseases [36]. Among patients receiving sacubitril/valsartan, three very rare cases of psychotic symptoms have been reported. All affected individuals were men of different ages and with varying baseline left ventricular ejection fractions. In these cases, the symptoms appeared shortly after initiation of therapy or after a dose increase, and resolved following discontinuation of or dose reduction in the drug. This observation suggests that the events may represent a rare adverse drug reaction. However, analysis of a larger patient population did not reveal additional similar incidents, indicating that this phenomenon is exceptionally uncommon. Therefore, if any concerning psychiatric symptoms occur during therapy, they should be promptly reported to a physician [37].

### 3.3. QTc

In clinical practice, QTc values > 450 ms in men and >470 ms in women should be considered abnormal, while QTc ≥ 500 ms or an increase of ≥60 ms from baseline should be regarded as clinically significant and warrant prompt evaluation of reversible factors, medication review, and consideration of treatment modification.

In the context of cardiovascular safety in patients with HF, monitoring the QTc interval is crucial, as its prolongation increases the risk of serious arrhythmias, including torsades de pointes (TdP). Among antipsychotic medications, the highest risk of QTc prolongation has been observed with thioridazine, intravenous haloperidol, and ziprasidone. Available evidence indicates that aripiprazole has a more favorable safety profile with regard to its effect on ventricular repolarization. Although SGAs are generally considered cardiologically safer, ziprasidone represents a notable exception. The mechanism underlying this effect is related to blockade of the IKr potassium channel (the delayed rectifier current) in cardiomyocytes, which prolongs repolarization and thereby increases the risk of cardiac rhythm disturbances [38]. Among antipsychotic medications, there is considerable heterogeneity in the risk of QTc interval prolongation, which has important implications for the safety of pharmacotherapy. Ziprasidone, iloperidone, and sertindole exhibit a significantly higher proarrhythmic potential compared with agents such as brexpiprazole, cariprazine, olanzapine, or even clozapine. Emerging real-world data further support the favorable cardiovascular tolerability of brexpiprazole; a clinical study by Martiadis et al. conducted in an Italian sample of psychiatric patients, including those with treatment-resistant presentations and relevant metabolic comorbidities, provided safety and tolerability data consistent with its low proarrhythmic potential [39]. Particularly high risk has been associated with the use of sertindole and ziprasidone, which belong to the group of drugs most strongly linked to this complication. In contrast, lurasidone represents a therapeutic option with a relatively more favorable cardiovascular safety profile. This observed pattern can be explained by a key molecular mechanism: the risk of QTc prolongation is directly proportional to a drug’s affinity for the cardiac hERG potassium channel. The greater the affinity, the stronger the blockade of ventricular repolarization, and consequently the higher the likelihood of cardiac arrhythmias [40].

Thirteen active substances were evaluated, including drugs commonly used in patients with HF (atenolol, digoxin, ramipril, simvastatin, calcium carbonate) as well as psychotropic medications (clozapine, olanzapine, quetiapine, ziprasidone), with regard to their association with undesirable QT interval shortening. Despite reported cases, the analysis did not confirm a clear causal relationship between any of the investigated drugs and QT shortening. Although QT interval shortening has also been discussed as a potential electrophysiological concern, current evidence remains limited and its clinical relevance in patients with HF is far less established than that of QTc prolongation. However, it should be emphasized that from a pharmacological safety perspective, particularly in vulnerable populations such as patients with HF, current testing requirements do not completely exclude a potential proarrhythmic risk associated with this phenomenon. This concern applies to both cardiovascular drugs and psychotropic medications, which are often used concomitantly in clinical practice [41]. Further studies are therefore necessary, taking into account not only the duration but also the morphology of the cardiac action potential, in order to fully assess the proarrhythmic safety profile of this group of drugs.

The use of antipsychotic medications in patients with HF to control agitation associated with delirium has been associated with a significant risk of cardiotoxicity. Such pharmacotherapy may induce QTc interval prolongation, which represents an important risk factor for potentially life-threatening ventricular arrhythmias, such as torsades de pointes (TdP). This risk is cumulative in cardiac patients, who frequently present with additional proarrhythmic factors, including electrolyte disturbances or structural myocardial damage [42]. The therapeutic decision should be based on an individual assessment of the benefit–risk balance, taking into account the dangers of uncontrolled delirium versus the risk of arrhythmia. A QTc interval > 500 ms requires re-evaluation of the treatment, unless its modification would pose a greater immediate threat to the patient.

In the context of managing the risk of QT interval prolongation, drug selection is crucial. In the treatment of psychotic symptoms, preference may be given to dopamine and serotonin receptor antagonists with minimal effects on ventricular repolarization, including lurasidone, aripiprazole, paliperidone, and asenapine. At the same time, the use of agents with a high proarrhythmic potential—such as amisulpride, ziprasidone, iloperidone, risperidone, olanzapine, quetiapine, or haloperidol—should be avoided.

An essential component of management is close electrocardiographic monitoring, including mandatory assessment of the corrected QT interval (QTc) [43].

Haloperidol induces QT interval prolongation through specific blockade of the rapid delayed rectifier potassium channels (IKr), which are crucial for cellular repolarization, thereby leading to prolongation of the cardiomyocyte action potential. This effect is dose-dependent, resulting in an average QT prolongation of 15–30 ms, which places this drug in the high-risk group for proarrhythmia. The most serious clinical manifestation of this electrophysiological dysfunction is the potential induction of life-threatening polymorphic ventricular tachycardia, torsades de pointes (TdP). Therefore, the use of haloperidol requires particular caution, especially in the context of polypharmacy with other QT-prolonging drugs or in conditions that predispose to arrhythmias, such as hypokalemia [44].

## 4. Beta-Adrenergic Blockers

β-blockers are a heterogeneous group of drugs whose pharmacodynamic properties determine their clinical profile and safety. According to current guidelines, β-blockers are not first-line agents in the treatment of hypertension [45]; however, as adjunctive therapy, they improve blood pressure control and reduce the effects of excessive sympathetic activity, particularly after myocardial infarction and in HF. Cardioselectivity reduces the risk of adverse effects associated with β2-receptor blockade [46]. Beta-blockers differ in their lipophilicity, which determines their pharmacokinetics and adverse effect profile. Lipophilic agents (e.g., pindolol, propranolol, metoprolol) are metabolized in the liver and more frequently produce central nervous system effects. Hydrophilic agents (e.g., atenolol, sotalol) are excreted by the kidneys and exhibit a more predictable pharmacological effect, whereas agents with intermediate lipophilicity (e.g., bisoprolol) are eliminated via dual pathways, which enhances their clinical safety [47].

### 4.1. Interactions of Mood Stabilizers with Beta-Blockers (Table 1)

In available pharmacokinetic analyses, β-blockers have been classified as drugs that may potentially impair lithium clearance. This mechanism may result from their effect on reducing renal blood flow, similarly to the action of other cardiovascular drugs (e.g., calcium channel blockers) [48]. β-adrenergic blockers may mask early symptoms of lithium toxicity, such as tachycardia or hypertension, due to their bradycardic effect, which can significantly hinder and delay the recognition of lithium toxicity. For this reason, current therapeutic recommendations for the treatment of HF advise particular caution when lithium and β-blockers are used concomitantly. Such a therapeutic combination requires increased clinical vigilance and regular assessment of serum lithium concentrations in order to prevent lithium accumulation and the development of toxic symptoms [48].

The concomitant use of CBZ, a strong inducer of hepatic CYP3A4 enzymes, with beta-blockers metabolized in the liver (e.g., propranolol, metoprolol) may potentially lead to accelerated metabolism of these drugs. As a consequence, this may result in reduced plasma concentrations and a possible weakening of the beta-blocker’s therapeutic effect. In such situations, dose adjustment (increase) of the beta-blocker may be required. This interaction does not significantly affect beta-blockers primarily excreted by the kidneys (e.g., atenolol, nadolol), and among those metabolized in the liver, it has not been clearly confirmed in clinical studies [49].

The available scientific literature reveals a clear research gap regarding the safety and efficacy of the concomitant use of mood stabilizers—such as valproic acid, carbamazepine, and lamotrigine—with beta-adrenergic receptor blockers in patients with diagnosed HF. Current literature in this pharmacological context focuses almost exclusively on patients with migraine, in whom this combination is sometimes used for prophylactic purposes. There is a lack of specialized analyses, clinical trials, or clinical guidelines that systematically evaluate the potential pharmacodynamic and pharmacokinetic interactions, as well as the overall impact of such polytherapy on clinical status, hemodynamics, and prognosis in patients with impaired systolic or diastolic myocardial function. Addressing this gap is particularly important given the potentially unfavorable effects of some antiepileptic drugs on cardiac function and the widespread use of beta-blockers as one of the pillars of HF treatment.

### 4.2. Interactions of Antipsychotic Drugs with Beta-Blockers (Table 1)

Scientific studies indicate a significant risk of interactions between β-adrenergic blockers and antipsychotic medications, particularly affecting the cardiovascular system. The concomitant use of risperidone and atenolol has been associated with the occurrence of atrial fibrillation. Clinical reports have also described bradycardia in patients receiving perphenazine together with metoprolol, as well as cases of ventricular arrhythmias following the combined administration of ziprasidone and sotalol. These observations suggest the presence of potentially unfavorable pharmacodynamic interactions between these drug combinations. Additionally, interactions between chlorprothixene and metoprolol or nebivolol may lead to enhanced hypotension, resulting from the synergistic blood pressure-lowering effects of these medications [50].

Other studies highlight the potential for pharmacokinetic interactions between beta-blockers and antipsychotics, particularly those related to metabolism via the CYP2D6 isoenzyme. Inhibitors of this enzyme may lead to increased plasma concentrations of both beta-blockers and antipsychotic drugs, which in turn may increase the risk of adverse effects [51].

## 5. Mineralocorticoid Receptor Antagonists (MRAs)

The pathophysiological activation of the mineralocorticoid receptor (MR) plays a key role in the development of cardiovascular diseases, such as HF and chronic kidney disease. Although MRAs, such as spironolactone and eplerenone, effectively reduce mortality, their use is limited due to the risk of hyperkalemia and deterioration of renal function, particularly when combined with RAAS inhibitors [52]. The new non-steroidal third-generation MRA, finerenone, is characterized by a more favorable safety profile, with a significantly lower risk of the above adverse effects, as demonstrated in phase II clinical trials in patients with HF and concomitant kidney disease or diabetes [53].

### 5.1. Interactions of Mood Stabilizers with MRAs (Table 1)

The concomitant use of spironolactone and lithium has been associated with a high risk of a clinically significant drug interaction and requires particular caution and close monitoring of serum lithium levels. The mechanism of this interaction involves a reduction in the renal clearance of lithium caused by spironolactone, which may lead to lithium accumulation and an increased risk of toxic effects. In situations where such a therapeutic combination is unavoidable, individual dose adjustment and regular follow-up of lithium concentrations and liver function parameters is advisable [54].

In the context of pharmacological interactions, the potential co-administration of spironolactone and VPA requires caution. Both drugs are metabolized in the liver, and spironolactone may inhibit cytochrome P450 enzymes, potentially increasing VPA concentrations and the risk of its adverse effects, such as somnolence, tremor, or hepatotoxicity. Additionally, the synergistic effects of these drugs may enhance sedation, which could negatively affect cognitive function [55,56]. Therefore, monitoring of VPA serum levels and liver function parameters is necessary during combined therapy.

Carbamazepine and spironolactone are recognized inducers of the intestinal transporter protein MRP2, acting through activation of the pregnane X receptor (PXR). Theoretically, their concomitant administration may lead to additive induction of this transporter, potentially accelerating efflux into the intestinal lumen and reducing the bioavailability of these drugs themselves or of other concurrently administered substances that are MRP2 substrates. However, the clinical significance of this pharmacokinetic interaction remains uncertain, and it may be masked by the concurrent induction of drug-metabolizing enzymes, such as CYP3A4 [57].

In the scientific literature, there are no reports describing the concomitant use of MRAs with LTG, and the potential metabolic pathways do not indicate the presence of unfavorable pharmacokinetic or pharmacodynamic interactions between these drugs.

### 5.2. Interactions of Antipsychotic Drugs with MRAs (Table 1)

In a double-blind, randomized clinical trial involving 46 patients with schizophrenia, the efficacy of adding spironolactone to risperidone therapy was evaluated. After the exclusion of six participants (due to premature withdrawal), the final analysis included 40 patients who completed the 8-week treatment period. At baseline, the groups (risperidone + spironolactone vs. risperidone + placebo) did not differ in symptom severity measured by the PANSS scale. Both groups demonstrated significant clinical improvement (*p* < 0.001). However, the spironolactone group achieved significantly greater reductions in negative symptoms (*p* = 0.004), positive symptoms (*p* = 0.007), and the total PANSS score (*p* = 0.042). A two-way ANOVA confirmed a significant time × intervention interaction for negative symptoms (*F* = 9.04; *p* = 0.002), positive symptoms (*F* = 3.43; *p* = 0.023), and the total PANSS score (*F* = 3.94; *p* = 0.022). However, no significant effect was observed on the general psychopathology subscale [58].

The concomitant use of typical antipsychotics and mineralocorticoid receptor antagonists (MRAs) is not generally considered a source of clinically significant pharmacokinetic or pharmacodynamic interactions. However, the potential for additive adverse effects should be taken into account, such as QT interval prolongation (e.g., with haloperidol) and hyperkalemia (associated with spironolactone). These considerations arise mainly from the known safety profiles of the individual drugs, rather than from evidence obtained in prospective clinical studies.

## 6. SGLT2 Inhibitors (Flozins)

Sodium–glucose cotransporter-2 (SGLT2) inhibitors, also known as flozins, are a class of antihyperglycemic drugs whose mechanism of action involves inhibition of glucose reabsorption in the renal tubules, leading to increased urinary glucose excretion (glucosuria) and a reduction in blood glucose levels. Beyond their glycemic benefits, drugs from this group exert significant cardio- and nephroprotective effects, as demonstrated in clinical trials, and they also contribute to weight reduction and lower blood pressure. Their metabolism occurs mainly through glucuronidation mediated by UGT enzymes, which makes them drugs with a relatively low potential for clinically significant drug–drug interactions compared with substances metabolized via the cytochrome P450 system [59]. In preclinical studies conducted in rats receiving an infusion of phlorizin, a classical sodium–glucose cotransporter inhibitor, a significant increase in the fractional excretion of glucose was observed, accompanied by an increase in creatinine clearance and renal lithium clearance. Despite the increased creatinine clearance, plasma creatinine concentrations remained unchanged, suggesting no acute impairment of glomerular filtration function. At the same time, a reduction in plasma lithium concentration was observed, indicating increased renal excretion of lithium [60]. SGLT2is block the sodium–glucose cotransporter SGLT2 in the proximal renal tubule, which reduces the reabsorption of both glucose and sodium in this segment of the nephron. As a result, natriuresis (sodium loss in the urine) and osmotic diuresis (excretion of water in the urine due to glucosuria) occur, which may lead to a reduction in body fluid volume [61].

### 6.1. Interactions of Mood Stabilizers with SGLT2 Inhibitors (Flozins) (Table 1)

Empagliflozin reduces the reabsorption of not only sodium and glucose but also lithium in the proximal renal tubules, which may lead to increased urinary excretion of lithium. As a consequence, serum lithium concentrations may decrease below the therapeutic range, potentially increasing the risk of relapse of psychiatric symptoms in patients stabilized with lithium therapy [62,63]. These observations indicate a significant pharmacological interaction between empagliflozin and lithium, while also confirming the role of the SGLT2 transporter in the physiological regulation of lithium handling. Moreover, these findings suggest a potential therapeutic application of SGLT2 inhibitors in the management of severe lithium toxicity, as agents capable of increasing renal lithium clearance. Although the strong effect of empagliflozin on serum lithium concentrations has not been well documented previously, this relationship has a clear theoretical basis. Lithium is reabsorbed primarily via sodium transporters in the proximal renal tubule, whereas empagliflozin inhibits the sodium–glucose cotransporter (SGLT2) in the same segment of the nephron. The interaction between these mechanisms may explain the observed pharmacological interaction [63]. SGLT2 inhibitors significantly reversed the negative trajectory of eGFR decline in patients receiving long-term lithium therapy, changing the mean annual rate of change from a significant decline (−1.43 mL/min/1.73 m^2^/year) to an increase (+0.69 mL/min/1.73 m^2^/year). The observed beneficial effect, corresponding to a total change of +2.13 mL/min/1.73 m^2^/year, suggests a potential nephroprotective role of these drugs in lithium-induced nephropathy. Despite these promising findings, randomized controlled trials are necessary to confirm the efficacy and to determine the therapeutic role of SGLT2 inhibitors in this specific clinical population [64].

SGLT2 inhibitors, by inducing weight reduction, may significantly affect the pharmacokinetics of drugs with a narrow therapeutic index, such as VPA. In the reported case, weight loss associated with empagliflozin therapy likely contributed to an increase in VPA serum concentration, which manifested as symptoms of neurotoxicity. Therefore, in patients initiating SGLT2 inhibitor therapy, enhanced monitoring of serum concentrations of drugs sensitive to weight-dependent distribution changes is recommended [65]. Monitoring serum VPA concentrations and adjusting its dosage after initiation of empagliflozin therapy are essential to prevent iatrogenic toxicity.

Based on the current literature and reliable drug-interaction databases, no clinically significant interactions have been identified between SGLT2 inhibitors and CBZ or LTG, and therefore dose modification of these agents is generally not required.

### 6.2. Interactions of Antipsychotic Drugs with SGLT2 Inhibitors (Flozins) (Table 1)

Atypical antipsychotics are associated with a broad spectrum of metabolic adverse effects—including weight gain, insulin resistance, and dyslipidemia—that collectively contribute to a substantial cardiometabolic burden in patients with schizophrenia and related disorders, which has important implications for long-term cardiovascular risk management [66]. In one systematic review, based on the available evidence—including one preclinical study, two clinical guideline recommendations, one systematic review, and one case report—an analysis of their conclusions was conducted. The use of SGLT2 inhibitors in patients with type 2 diabetes treated with antipsychotics such as olanzapine or clozapine is partially supported by their favorable metabolic profile. In selected cases, combination therapy with metformin may represent a purposeful therapeutic strategy. However, the recommendation positioning this class of drugs as second-line therapy in this specific clinical population is based on very limited evidence, derived mainly from preliminary preclinical studies and a small number of clinical observations [67]. Despite their potential to mitigate the metabolic adverse effects of atypical antipsychotics, direct evidence confirming the efficacy and safety of SGLT2 inhibitors in combination with olanzapine or clozapine remains insufficient. This highlights the urgent need for further well-designed studies in this area [68].

According to available data, SGLT2 inhibitors may, in rare cases, induce the development of ketoacidosis with normal blood glucose levels (euglycemic ketoacidosis). Therefore, particular caution is recommended in patients who are concurrently receiving antipsychotic medications that may also have ketogenic potential, such as olanzapine and risperidone [69,70]. The assessment of the risk of this complication should take into account the significant cardiometabolic benefits associated with the use of SGLT2 inhibitors, including their proven cardioprotective and nephroprotective effects in selected patient populations [69].

## 7. Diuretics in HF—Symptomatic Treatment

Diuretics are essential in the treatment of hypervolemia in patients with HF, and their mechanism of action involves increasing the excretion of water and sodium, thereby reducing cardiac workload. Examples include furosemide and torasemide, which effectively lower BNP levels, while torasemide additionally reduces cardiac fibrosis and edema. However, their use has been associated with a risk of adverse effects, mainly disturbances in electrolyte and acid–base balance, which may predispose patients to serious arrhythmias [71].

### 7.1. Interactions of Mood Stabilizers with Diuretics (Table 1)

Any clinical conditions leading to hyponatremia may induce an increase in serum lithium concentrations to the toxic range. Diuretics that reduce renal sodium reabsorption and increase its urinary excretion—such as thiazides, aldosterone antagonists, and sodium channel inhibitors—are particularly associated with lithium accumulation. A similar mechanism of elevated lithium levels is observed in cardiac patients with impaired renal perfusion. In contrast, osmotic diuretics, which improve glomerular filtration, may lower lithium concentrations. It is also worth noting that peripheral edema (e.g., ankle edema), a known adverse effect of lithium, may be managed with appropriate diuretic therapy. In summary, interactions between lithium and drugs affecting the RAAS as well as sodium–water homeostasis require particular clinical attention and careful monitoring of lithium serum concentrations [72]. The assessment of the pharmacokinetic interaction between thiazide diuretics and lithium represents an important clinical issue in the treatment of psychiatric disorders. The concomitant administration of these agents may require consideration with a high risk, as it may lead to a significant reduction in the renal clearance of lithium. The primary mechanism of this pharmacological phenomenon involves inhibition of lithium excretion in the renal tubules, which results directly from increased reabsorption of lithium in the distal segments of the nephron. Consequently, lithium accumulates in the body, leading to serum concentrations exceeding the safe therapeutic range. This significantly increases the risk of toxic manifestations, including neurological, gastrointestinal, and cardiovascular symptoms [21].

The use of thiazide diuretics requires particular caution, as by reducing lithium clearance they may significantly increase serum lithium concentrations, thereby raising the risk of lithium toxicity. In contrast, amiloride is considered a safer diuretic option, as it blocks the entry of lithium into renal tubular cells, thereby reducing its accumulation. However, it should be noted that other diuretics, such as loop diuretics or potassium-sparing agents, may also affect lithium concentrations. Therefore, careful monitoring of serum lithium levels is required in all cases [20].

The literature describes a case of a 60-year-old woman who developed symptomatic hyponatremia after CBZ was added to ongoing hydrochlorothiazide therapy. The symptoms resolved and serum sodium levels normalized after the thiazide diuretic was replaced with a beta-blocker, while CBZ therapy was continued. This observation suggests an additive adverse effect of both drugs on lowering serum sodium levels, which should be taken into account when prescribing them concomitantly [73,74]. In a study comparing the risk of hyponatremia, oxcarbazepine was found to be associated with a more than tenfold higher risk of developing this complication (1.661% of patients) compared with CBZ (0.169% of patients) [75].

Based on the current literature and reliable drug-interaction databases, no clinically significant interactions have been identified between diuretics and VPA or LTG, which does not indicate the need for dose modification of these drugs.

### 7.2. Interactions of Antipsychotic Drugs with Diuretics (Table 1)

In the population of patients with HF, in whom diuretic therapy is routinely used, an important issue is the precise assessment of the iatrogenic risk of hyponatremia in the context of polypharmacotherapy. Current evidence suggests that antipsychotic pharmacotherapy may represent a significant and modifiable risk factor for the development of this complication. Results from prospective cohort studies indicate substantial differences in the hyponatremia-inducing potential among various classes of psychotropic drugs. Epidemiological analyses have shown that the use of first-generation (typical) antipsychotics is associated with a significantly higher relative risk of hospitalization due to severe hyponatremia compared with therapy using SGAs. Importantly, within the SGA group, some agents have been identified as having a neutral safety profile regarding the risk of sodium balance disturbances—particularly risperidone and aripiprazole. Their use, both during treatment initiation and continuation, was not associated with a significant increase in hospitalization rates due to hyponatremia [76].

The use of antipsychotic medications in patients with HF may require consideration of significant cardiovascular risk, primarily due to QTc interval prolongation (observed in more than 10% of treated patients), which may lead to life-threatening ventricular arrhythmias such as torsades de pointes (TdP) and sudden cardiac death. This risk is particularly relevant in this patient population because of the widespread use of diuretic therapy. Diuretics, by inducing hypokalemia and hypomagnesemia, destabilize myocardial repolarization, acting synergistically with antipsychotics that block the hERG potassium channel. This combination creates a strongly proarrhythmic environment. Therefore, initiating antipsychotic therapy requires careful risk stratification, including baseline ECG assessment, regular monitoring of electrolyte levels, and a critical evaluation of the necessity of ongoing diuretic therapy [77]. Loop diuretics, by inducing hypokalemia, act synergistically with antipsychotic drugs (such as quetiapine and haloperidol), thereby increasing the risk of QT interval prolongation. Therefore, strict electrocardiographic monitoring and regular assessment of electrolyte levels are essential in these patients, and therapeutic decisions must take into account this potentially life-threatening interaction [78].

To facilitate clinical use, Table 1 provides a cross-referenced summary of interactions between psychotropic agents and heart failure therapies, allowing rapid identification of clinically relevant combinations across drug classes.

## 8. Discussion

Patients with HF who are also under psychiatric care and receive both antipsychotic and antihypertensive drugs simultaneously require strict monitoring due to the risk of weakening or enhancing the effect of either of these drugs. In order to minimize the risk of adverse effects, the combination of beta-blockers such as propranolol or pindolol with thioridazine or chlorpromazine should be avoided where possible. Relatively safest among these combinations can be considered antihypertensive drugs without central action, for example diuretics [79].

While ACEIs and ARBs affect lithium primarily by altering glomerular hemodynamics and reducing GFR, ARNIs introduce an additional mechanism—neprilysin inhibition. The resulting increase in natriuretic peptide concentrations (ANP, BNP) promotes natriuresis (urinary sodium excretion) [80]. In accordance with renal physiology, the body in a state of hyponatremia compensatorily increases tubular reabsorption in the proximal tubule, where lithium is handled by transporters in a manner nearly identical to sodium. This mechanism is supported by the literature, which indicates that natriuresis induced by natriuretic peptides (ANP, BNP) may activate compensatory mechanisms in the proximal renal tubule. In line with tubular transport physiology, increased sodium reabsorption in this nephron segment is inseparably linked to increased lithium ion reabsorption, which—in the context of ARNI therapy—constitutes an additional, RAAS-independent risk factor for lithium toxicity [81,82].

Mood stabilizers such as lithium and VPA are widely used in affective disorders, but their use in patients with heart failure (HF) requires careful consideration. Lithium is of particular concern because of its potential to induce conduction abnormalities, including sinus bradycardia and atrioventricular block, especially in patients with underlying sinus node dysfunction [1,83]. VPA is generally not associated with major direct cardiovascular interactions, although thrombocytopenia and platelet dysfunction may still be clinically relevant in selected HF patients [84].

The cardiac safety concerns associated with LTG, including FDA communications regarding arrhythmia risk in patients with HF, are discussed in detail in Section 3.1.5.

From a practical perspective, the interactions discussed in this review should be interpreted within the framework of contemporary guideline-directed HF therapy. In routine care, psychotropic drug selection should not be considered separately from the standard pharmacological pillars of HF management, but rather integrated with them. This is especially important in patients receiving multidrug regimens that may influence renal function, electrolyte balance, hemodynamic stability, or ventricular repolarisation.

The evidence discussed in this review is heterogeneous and includes preclinical studies, case reports, observational analyses, and selected clinical studies. Consequently, some reported associations should be interpreted with caution and viewed as hypothesis-generating rather than definitive.

To improve clinical applicability, we summarized the key interaction patterns in a structured format integrating patient context, mechanisms, and potential biomarkers (Supplementary Table S1).

## 9. Strengths and Limitations

A major strength of this review is its clinically integrated perspective on psychopharmacological treatment in patients with HF, a population in whom polypharmacy, renal vulnerability, electrolyte instability, and arrhythmic risk frequently overlap. By organizing the literature around contemporary guideline-directed HF therapies, this review aims to provide a practical framework for clinicians working at the interface of cardiology and psychiatry.

This review focuses on mood stabilisers and antipsychotics that may be used in patients with HF. Antidepressants are, of course, also widely prescribed in this population. Their omission may therefore appear as a limitation; however, this topic will be addressed in a separate article due to publication constraints.

However, several limitations must be acknowledged. This review was conducted as a narrative synthesis rather than a formal systematic review or meta-analysis. This approach was chosen deliberately, as a number of systematic reviews addressing related questions are already available, while our primary objective was to emphasise clinically relevant insights and practical implications for the management of patients with HF. Consequently, the body of evidence discussed is inherently heterogeneous, comprising case reports, pharmacokinetic observations, preclinical studies, observational analyses, and selected clinical reports rather than large prospective HF-specific trials. The inclusion of case-based evidence was intentional, as such reports often provide important mechanistic insights and highlight clinically relevant interactions that remain underrepresented in larger studies.

As a result, causal inference is limited, and the presence or absence of isolated reports should not be interpreted as a direct measure of real-world incidence. Moreover, for many specific psychotropic–HF drug combinations, robust frequency estimates are lacking, and it remains unclear how risk varies across clinically important subgroups such as older adults, patients with chronic kidney disease, frailty, or different ethnic backgrounds. Therefore, the interactions discussed in this review should be interpreted primarily as risk signals of variable evidentiary strength rather than uniformly established clinical facts.

For this reason, the main contribution of the present work is not to quantify incidence, but to provide a mechanistically and clinically structured interpretation of the interactions most relevant to everyday care.

## 10. Take-Home Messages

1. The use of lithium with RAAS inhibitors (ACEi, ARBs, ARNi), thiazide diuretics, and SGLT2 inhibitors requires mandatory monitoring of drug levels and renal parameters; changes in water–electrolyte metabolism caused by these drug groups drastically increase the risk of lithium toxicity.
2. In patients with HF requiring diuretics, the use of SGAs (especially risperidone and aripiprazole) is safer than first-generation agents; however, each case requires prophylactic correction of electrolyte disturbances (hypokalemia, hypomagnesemia) to minimize arrhythmia risk.
3. In the population of patients with HF, lamotrigine and levetiracetam represent safer alternatives to valproic acid, whose use is associated with a statistically significant increase in mortality risk.
4. Addition of spironolactone to standard risperidone treatment may represent an effective strategy for improving control of both positive and negative symptoms of schizophrenia.
5. Concurrent use of beta-blockers with neuroleptics requires enhanced clinical vigilance due to the risk of synergistic, adverse effects on hemodynamic parameters and the cardiac conduction system.

## Figures and Tables

**Table 1:** Summary of pharmacological interactions between the HF drugs and antipsychotics and mood stabilizers.

Drug Group (Psychiatric/ Cardiological)	Atypical Antipsychotics/SGAs	Typical Antipsychotics/FGAs	Lithium	Valproic Acid (VPA)	Carbamazepine (CBZ)	Lamotrigine (LTG)
**Angiotensin-converting enzyme inhibitors (ACEIs)**	Orthostatic hypotension	Orthostatic hypotension (particularly chlorpromazine and thioridazine)	Lithemia ↑ (reduced clearance) Mechanism: altered renal hemodynamics and reduced lithium excretion. More frequent Li monitoring and dose adjustment required.	No clinically significant interactions Enalapril enhances the anticonvulsant effect of valproate.	possible attenuation of cardiac drug therapeutic effect	Enalapril enhances anticonvulsant activity; cilazapril does not produce this effect
**Angiotensin receptor blockers (ARBs)**	Orthostatic hypotension	Orthostatic hypotension (particularly chlorpromazine and thioridazine)	Lithemia ↑ (reduced clearance), hyperkalemia, hyponatremia	No clinically significant interactions	possible attenuation of cardiac drug therapeutic effect	No clinically significant interactions
**ARNI (sacubitril /valsartan)**	Orthostatic hypotension	Orthostatic hypotension (particularly chlorpromazine and thioridazine)	Lithemia ↑ (reduced clearance)	No clinically significant interactions	possible attenuation of cardiac drug therapeutic effect	No clinically significant interactions
**Beta-blockers**	Bradycardia, atrial fibrillation (risperidone + atenolol), and ventricular arrhythmia (ziprasidone + sotalol)	Bradycardia (perphenazine + metoprolol), hypotension (chlorprothixene + metoprolol/nebivolol)	Masking of toxicity symptoms + lithemia ↑ (reduced clearance)	*No clinically significant pharmacodynamic interactions* *Additional sedation may occur.*	Potentially decreases the concentrations of β-blockers metabolized by CYP enzymes → weakening of the therapeutic effect	No clinically significant interactions
**Aldosterone antagonists**	Possible hyperkalemia and QTc prolongation. Better control of positive and negative symptoms with spironolactone augmentation compared with placebo (in combination with risperidone).	Hyperkalemia and QTc prolongation (as independent adverse effects of both drug classes)	Hyperkalemia + lithium toxicity (monitor K^+^ and Li^+^)—reduced lithium clearance	No clinically significant interactions	Enzyme induction may reduce plasma levels of CYP-metabolized MRAs	No clinically significant interactions
**SGLT2 inhibitors**	Risk of ketoacidosis	No clinically significant interactions	Risk of subtherapeutic lithium levels; beneficial in managing lithium toxicity	Risk of VPA neurotoxicity due to excessive dehydration	Possible reduced SGLT2 inhibitor exposure due to carbamazepine-mediated MRP2 induction	No clinically significant interactions
**Diuretics (e.g., furosemide, torasemide)**	Hyperglycemia + dehydration + QTc prolongation	Dehydration + QTc prolongation	Lithemia ↑ (osmotic diuresis) + hyponatremia Thiazides markedly may increase lithium concentration levels (mechanism: reduced sodium excretion → increased lithium reabsorption) Loop diuretics have a smaller but still significant effect (dehydration → ↑Li). Loop diuretic use requires Li and renal function monitoring	No clinically significant interactions	Additive lowering of serum sodium	Worsening of hypokalemia.

Red—high-risk interactions with potential for serious or life-threatening adverse effects (e.g., arrhythmia, lithium toxicity). Yellow—interactions of potential but context-dependent clinical relevance, requiring clinical awareness and individualized monitoring. Green—no clinically significant interaction or generally safe combination based on current evidence. Abbreviations: ACEIs—angiotensin-converting enzyme inhibitors; ARBs—angiotensin receptor blockers; ARNI—angiotensin receptor–neprilysin inhibitor; MRAs—mineralocorticoid receptor antagonists; SGLT2—sodium–glucose cotransporter-2 inhibitors; QTc—corrected QT interval; ↑—increase level; →—consequences.

## Data Availability

No new data were created or analyzed in this study. Data sharing is not applicable to this article.

## Supplementary Materials

The following supporting information can be downloaded at: https://www.mdpi.com/article/10.3390/brainsci16050452/s1. Table S1. The key aspects of the discussed drug interactions.
