# Supplementary material for: Psychopharmacological Treatment in Patients with Heart Failure: A Narrative Review of Mood Stabilizers and Antipsychotics
Source: Brain Sci. 2026 Apr 24;16(5):452. doi: 10.3390/brainsci16050452 (PMC13204832; doi:10.3390/brainsci16050452)
Supplement: Supplementary file 1 [file brainsci-16-00452-s001.zip › brainsci-4235454-supplementary.pdf]

Supplementary Table S1. The key aspects of the discussed drug interactions.

Drug combination	Typical patient profile	Clinical context	Proposed mechanism	Common biological pathway	Main adverse effects	Suggested biomarkers / monitoring
**Lithium + ACEI/ARB/ARNI**	Older adults, HF patients, CKD, polypharmacy	HF + hypertension, renal vulnerability	↓ renal lithium clearance due to altered renal hemodynamics and sodium balance	RAAS inhibition → ↓ GFR / natriuresis → ↑ proximal lithium reabsorption	Lithium toxicity (confusion, tremor, arrhythmia), AKI	Serum lithium, creatinine, eGFR, sodium, potassium
**Lithium + thiazide diuretics**	Elderly, volume-depleted patients	HF with fluid overload	↑ lithium reabsorption in distal nephron due to sodium depletion	Sodium–lithium co-transport in renal tubules	Severe lithium toxicity	Serum lithium (frequent), sodium, volume status
**Lithium + MRAs (spironolactone)**	HF + CKD + RAAS blockade	Advanced HF	↓ lithium clearance, possible additive renal effects	RAAS + mineralocorticoid pathway	Lithium toxicity, hyperkalemia	Lithium, potassium, creatinine
**Lithium + SGLT2 inhibitors**	HF + diabetes, metabolic syndrome	HF + T2DM	↑ renal lithium excretion via proximal tubule inhibition	SGLT2-mediated sodium–glucose transport	Subtherapeutic lithium levels, relapse risk	Lithium levels, glycemia, renal function
**Antipsychotics + diuretics**	Elderly HF patients, frailty	HF + electrolyte disturbances	Indirect interaction via hypokalemia/hypomagnesemia	hERG channel blockade + electrolyte imbalance	QT prolongation, TdP, sudden cardiac death	ECG (QTc), potassium, magnesium
**Antipsychotics + beta-blockers**	Cardiovascular comorbidity	HF + arrhythmia risk	Additive effects on conduction and autonomic tone	β-adrenergic signaling + cardiac conduction pathways	Bradycardia, hypotension, arrhythmias	ECG, heart rate, blood pressure
**Antipsychotics + ACEI/ARB/ARNI**	Elderly, polypharmacy	HF + hypertension	Additive hypotensive effect	RAAS + autonomic regulation	Orthostatic hypotension, falls	Blood pressure (standing/supine), electrolytes
**Ziprasidone / haloperidol + HF therapy**	High-risk cardiac patients	HF + polypharmacy	Direct QT prolongation via hERG blockade	Cardiac repolarization (IKr channel)	QT prolongation, TdP	ECG (QTc), electrolytes

**Valproate + SGLT2 inhibitors**	Obese / metabolic patients	HF + T2DM	Indirect PK effect via weight loss and distribution changes	Metabolic pathways, hepatic metabolism	VPA toxicity (sedation, neurotoxicity)	VPA levels, liver function tests
**Carbamazepine + beta-blockers**	Polypharmacy patients	HF + arrhythmia	CYP3A4 induction → ↓ beta-blocker levels	Hepatic enzyme induction	Reduced efficacy of β-blockers	Drug levels (if available), BP, HR
**Lamotrigine (± HF therapy)**	HF patients with mood disorders	HF + arrhythmia risk	Possible sodium channel blockade in myocardium	Cardiac sodium channels	Conduction abnormalities, arrhythmia (rare)	ECG, PR/QRS interval
